# Modified Fractional Variational Iteration Method for Solving the Generalized Time-Space Fractional Schrödinger Equation

**DOI:** 10.1155/2014/964643

**Published:** 2014-09-04

**Authors:** Baojian Hong, Dianchen Lu

**Affiliations:** ^1^Faculty of Science, Jiangsu University, Zhenjiang, Jiangsu 212013, China; ^2^Department of Basic Causes, Nanjing Institute of Technology, Nanjing 211167, China

## Abstract

Based on He's variational iteration method idea, we modified the fractional variational iteration method and applied it to construct some approximate solutions of the generalized time-space fractional Schrödinger equation (GFNLS). The fractional derivatives are described in the sense of Caputo. With the help of symbolic computation, some approximate solutions and their iterative structure of the GFNLS are investigated. Furthermore, the approximate iterative series and numerical results show that the modified fractional variational iteration method is powerful, reliable, and effective when compared with some classic traditional methods such as homotopy analysis method, homotopy perturbation method, adomian decomposition method, and variational iteration method in searching for approximate solutions of the Schrödinger equations.

## 1. Introduction

In the past decades, due to the numerous applications of fractional differential equations (FDEs) in the areas of nonlinear science [1], many important phenomena can be described successfully using the FDEs models such as materials and processes [2], engineering and physics [3], dielectric polarization [4], and quantitative finance [5]. Searching for solutions of these FDEs plays an important and significant role in all aspects of this subject. But because of the complexity of nonlinear terms and fractional derivative, it is very difficult for us to obtain the exact analytic solutions of most FDEs, so approximate and numerical methods must be considered. A great deal of efforts have been proposed for these problems, like the homotopy analysis method (HAM) [6], the homotopy perturbation method (HPM) [7], the adomian decomposition method (ADM) [8], the generalized differential transform method [9], and so forth [10].

The variational iteration method (VIM) established in 1999 by He in [11] is thoroughly used by many researchers to construct the approximate solutions of a wide variety of scientific and engineering models [12, 13]. After some modifications, the fractional variational iteration method (FVIM) was applied to fractional differential equations by He and many authors [14–19]. The motivation of this paper is to construct some analytical approximate solutions for the GFNLS powerfully. Firstly, we give some modifications for the FVIM and extend the application of the FVIM. Secondly, we use the modified fractional variational iteration method (MFVIM) to the GFNLS and compare the efficiency of MFVIM with some other traditional perturbation methods. The results show that MFVIM gives rapid and standard convergence to the exact solution if such a solution exists.

We give some basic definitions and properties of the fractional calculus theory which are used further in this paper; we define the following fractional integral and derivatives [20, 21].

**Definition 1 .**

A real function *f*(*x*) is said to be in the space *C*_*μ*_, where *μ* ∈ *R*, *x* > 0, if there exists a real number *p*(>*μ*) such that *f*(*x*) = *x* ^*p*^ *f*_1_(*x*), where *f*_1_(*x*) ∈ *C*[0, *∞*) and it is said to be in the space *C*_*μ*_^*m*^ if and only if *f* ^(*m*)^ ∈ *C*_*μ*_, *m* ∈ *N*.

**Definition 2 .**

The Riemann-Liouville fractional integral operator of order *α* > 0 for a function *f*(*x*) ∈ *C*_*μ*_, *μ* ≥ −1, is defined as follows:

(1) Jxαf(x)=1Γ(α)∫0x(x−ξ)α−1f(ξ)dξ   =1Γ(α+1)∫0xf(ξ)(dξ)α, α>0,  x>0,Jx0f(x)=f(x).

Also one has the following properties:

(2) $$\begin{array}{c} J^{\alpha }J^{\beta }f\left(x\right)=J^{\alpha +\beta }f\left(x\right),\\ J^{\alpha }J^{\beta }f\left(x\right)=J^{\beta }J^{\alpha }f\left(x\right),\\ J^{\alpha }x^{\gamma }=\frac{\Gamma \left(\gamma +1\right)}{\Gamma \left(\alpha +\gamma +1\right)}x^{\alpha +\gamma }. \end{array}$$

**Definition 3 .**

For *α* > 0, *x* > 0, and *f*(*x*) ∈ *C*_−1_^*n*^, the Caputo fractional derivative operator of order *α* on the whole space is defined as follows:

(3) Dαf(x)=Jn−αDnf(x)={1Γ(n−α)∫0x(x−ξ)n−α−1f(n)(ξ)dξ,     n−1<α<n,  n∈N,d(n)f(x)dxn, α=n.

Also one has the following properties:

(4) DαC=0, (C  is  a  constant),Dαxγ={Γ(γ+1)Γ(γ−α+1)xγ−α,γ>α−1,0,γ≤α−1,JαDαf(x)=f(x)−∑k=0n−1f(k)(0+)xkk!,n−1<α<n,DαJαf(x)=f(x).

## 2. Analysis of the MFVIM and the FGNLS

Consider the following generalized time and space fractional nonlinear Schrödinger equation with variable coefficients [22, 23]:

(5) i∂αu∂tα+a∂2βu∂x2β+v(x)u+γ|u|2u=0,t>0, 0<α, β≤1,u(x,0)=f(x),

where *u* = *u*(*x*, *t*), ∂^*α*^ *u*/∂*t* ^*α*^ = *D*_*t*_^*α*^ *u*, ∂^2*β*^ *u*/∂*x* ^2*β*^ = *D*_*x*_^*β*^(*D*_*x*_^*β*^ *u*), *v*(*x*) is the trapping potential, and *a*, *γ* are the slowly increasing dispersion coefficient and nonlinear coefficient, respectively. If we select *α* = *β* = 1, *v*(*x*) = 0, this equation turns to the famous nonlinear Schrödinger equations in optical fiber [24–26].

According to the FVIM [14–19], we can build a correction functional for (5) as follows:

(6) un+1=un+1Γ(1+α)×∫0tλ(τ,x)[i∂αun∂tα+a∂2βu~n∂x2β      +v(x)u~n+γu~n|u~n|2](dτ)α,

with the initial condition *u*_0_ = *u*(*x*, 0) = *f*(*x*), where *λ*(*t*, *x*) is a general Lagrange's multiplier which can be identified optimally with the variational theory. The function ${\tilde{u}}_{n}$ is a restricted variation which means $\delta {\tilde{u}}_{n}=0$. Therefore, we first determine the Lagrange multiplier *λ*(*t*, *x*) that will be identified optimally via integration by parts [27]. The successive approximations *u*_*n*+1_, *n* ≥ 0, of the solution *u*(*x*, *t*) will be readily obtained through *λ*(*t*, *x*) and any selective function *u*_0_. The initial values are usually used for choosing the zeroth approximation *u*_0_. With *λ*(*t*, *x*) determined, then several approximations *u*_*k*_, *k* = 1,2,… follow immediately. Consequently, the exact solution may be procured by using *u* = lim⁡_*n*→*∞*_⁡*u*_*n*_. The convergence of FVIM has been proved in [28]. In this paper, notice that (5) is a complex differential equation with complex modulus term |*u*|^2^, as we all know, a complex function *u*(*ξ*) can be written as *c*(*ξ*)*e* ^*iθ*(*ξ*)^, where *c*(*ξ*) and *θ*(*ξ*) are real functions, noticed that |*u*(*ξ*)|^2^ = |*c*(*ξ*)|^2^, we can give some modification for the iteration formulation (6), assume that $\lim_{n\to \infty }{\left|{\tilde{u}}_{n}\right|}^{2}={\left|u\right|}^{2}={\left|u_{0}\right|}^{2}$, we get the MFVIM for (5). This modification should enhance rapidly the efficiency of our iteration.

In what follows, in order to illustrate the strength of this method, we will apply the MFVIM to some models about (5).

## 3. Approximate Solutions for the FGNLS

**Example 4 .**

We first consider the time-fractional NLS equation [29]:

(7) $$\begin{array}{c} i\frac{\partial^{\alpha }u}{\partial t^{\alpha }}+a\frac{\partial^{2}u}{\partial x^{2}}+\gamma u{\left|u\right|}^{2}=0,\;t>0,\;\;0<\alpha \leq 1,\\ u\left(x,0\right)=f\left(x\right). \end{array}$$

The correction functional for (7) reads

(8) un+1=un+1Γ(1+α)×∫0tλ(τ,x)[i∂αun∂tα+a∂2u~n∂x2+γu~n|u~n|2](dτ)α.

Making the above correction functional stationary,

(9) δun+1=δun+λ(t,x)iδun−1Γ(1+α)∫0t[iDτ(α)λ(τ,x)δun](dτ)α.

After getting the coefficients of *δu*_*n*_ to zero we can determine the Lagrange multiplier

(10) $$\begin{array}{c} \lambda =i. \end{array}$$

We produce the iteration formulation as follows:

(11) $$\begin{array}{c} u_{n+1}=u_{n}+\frac{i}{\Gamma \left(1+\alpha \right)}\overset{t}{\underset{0}{\int }}\left[i\frac{\partial^{\alpha }u_{n}}{\partial \tau^{\alpha }}+a\frac{\partial^{2}u_{n}}{\partial x^{2}}+\gamma u_{n}{\left|u_{0}\right|}^{2}\right]{\left(d\tau \right)}^{\alpha }. \end{array}$$

As stated before, we can select *u*_0_ = *u*(*x*, 0) = *A*sec *hx*; using the iteration (11) and the mathematica software, we obtain the following successive approximations:

(12) u0 =Asec hx,u1Asec hx+aAi(sec hx−2sec h3x)1Γ(1+α)tα +A3iγsec h3x1Γ(1+α)tα=Asec hx[1+aitαΓ(1+α)] +(−2a+A2γ)Aisec h3x1Γ(1+α)tα=Asec hx[1+aitαΓ(1+α)], (A2=2aγ),u2Asec hx+aAi(sec hx−2sec h3x) ×[1Γ(1+α)tα+ai1Γ(1+2α)t2α] +γA3isec h3x[1Γ(1+α)tα+ai1Γ(1+2α)t2α]=Asec hx[1+aitαΓ(1+α)+1Γ(1+2α)(aitα)2],u3 =Asec hx[1+aitαΓ(1+α)+(aitα)2Γ(1+2α)+(aitα)3Γ(1+3α)], ⋮un =Asec hx∑k=0n1Γ(1+kα)(aitα)k.

The exact solution of (7) is

(13) u=Asec hxlim⁡n→∞ ∑k=0n 1Γ(1+kα)(aitα)k=±2aγ sec hxEα(aitα),

where *E*_*α*_(*ait* ^*α*^) is the Mittag-Leffler function. If we let *α* = 1 in (13), the exact solution of the regular NLS equation (7) can be obtained as follows:

(14) $$\begin{array}{c} {u|}_{\alpha =1}=\pm \sqrt{\frac{2a}{\gamma }}\;\text{sec}\;hxe^{iat}. \end{array}$$

**Example 5 .**

We now consider the time-space fractional NLS equation [30, 31]:

(15) i∂αu∂tα+a∂2βu∂x2β+2au|u|2=0,t>0, 0<α, β≤1,u(x,0)=eix.

With the similar process, we get the iteration formulation as follows:

(16) un+1=un+iΓ(1+α)×∫0t[i∂αun∂τα+a∂2βun∂x2β+2aun|u0|2](dτ)α.

Using the iteration (16) and the mathematica software, we obtain the following successive approximations:

(17) u0 =eix,u1eix[1+(2+eiπβ)iatαΓ(1+α)]=eix(1+c1iatαΓ(1+α)), c1=2+eiπβ,u2eix{1+(2+eiπβ)iatαΓ(1+α)   +[(2+eiπβ)eiπβ    +2(2+eiπβ)]i2a2t2αΓ(1+2α)}=eix[1+c1iatαΓ(1+α)+c12i2a2t2αΓ(1+2α)],u3eix{1+(2+eiπβ)iatαΓ(1+α)   +(2c1+c1eiπβ)i2a2t2αΓ(1+2α)   +(2c12+c12eiπβ)i3a3t3αΓ(1+3α)}=eix[1+c1iatαΓ(1+α)+c12i2a2t2αΓ(1+2α)+c13i3a3t3αΓ(1+3α)], ⋮un =eix∑k=0n1Γ(1+kα)[i(2+eiπβ)atα]k.

The exact solution of (15) is

(18) u=eixlim⁡n→∞ ∑k=0n 1Γ(1+kα)[i(2+eiπβ)atα]k=eixEα[i(2+eiπβ)atα].

If we let *α* = 1 and let *β* = 1 in (18), the exact solution of the regular NLS equation (15) can be obtained as follows:

(19) $$\begin{array}{c} {u|}_{\left(\alpha =1,\beta =1\right)}=e^{i(x+at)}. \end{array}$$

**Remark 6 .**

The solution (18) is more standard than the result (3.18) in [30]. If one selects *a* = 1 or *a* = 1/2, the solution (19) is the same as the result (49) in [29], the result (3.21) in [30], and the result (29) in [31], but one can find that this iteration is much more standard and powerful than the HAM, the ADM, and the VIM mentioned in [29–31].

**Example 7 .**

Consider the following time-space fractional NLS equation [23]:

(20) i∂αu∂tα+12∂2βu∂x2β−ucos⁡2x−|u|2u=0,t>0, 0<α≤1, u(x,0)=sinx.

With the similar process, we get the iteration formulation as follows:

(21) un+1=un+iΓ(1+α)×∫0t[i∂αun∂τα+12∂2βun∂x2β−uncos⁡2x−un|u0|2](dτ)α.

If we select *u*_0_ = *u*(*x*, 0) = sin*x*, using the iteration (21) and the mathematica software, we obtain the following successive approximations:

(22) u0 =sinx,u1 =sinx+[12sin(x+πβ)−sinx]itαΓ(1+α),u2sinx+[12sin⁡(x+πβ)−sinx]itαΓ(1+α) +[14sin(x+2πβ)−sin(x+πβ)+sinx] ×i2t2αΓ(1+2α),u3u2+[18sin⁡(x+3πβ)−34sin⁡(x+2πβ)    +32sin(x+πβ)−sinx]i3t3αΓ(1+3α), ⋮un =∑k=0nck(x)(itα)kΓ(1+kα),

where *c*_*k*_(*x*) = *c*_*k*,0_sin*x* + *c*_*k*,1_sin(*x* + *πβ*) + *c*_*k*,2_sin(*x* + 2*πβ*)+⋯+*c*_*k*,*k*_sin(*x* + *k* *πβ*), *c*_*k*,0_ = (−1)^*k*^, *c*_*k*,1_ = (1/2)*c*_*k*−1,1_ − *c*_*k*−1,0_,…, *c*_*k*,*k*−1_ = (1/2)*c*_*k*−1,*k*−2_ − *c*_*k*−1,*k*−1_, and *c*_*k*,*k*_ = (1/2)*c*_*k*−1,*k*−1_, *k* ≥ 2, *c*_0,0_ = 1; *c*_1,0_ = −1, *c*_1,1_ = (1/2); *c*_2,0_ = 1, *c*_2,1_ = −1, *c*_2,2_ = (1/4); ….

The exact solution of (20) is

(23) u=lim⁡n→∞ ∑k=0n ck(x)(itα)kΓ(1+kα)=sin[xβΓ(1+β)]Exp[−32itαΓ(1+α)].

If we let *α* = 1 and let *β* = 1 in (23), the exact solution of the regular NLS equation (20) can be obtained as follows.

(24) $$\begin{array}{c} {u|}_{\left(\alpha =1,\beta =1\right)}=\sin xe^{-(3/2)it}. \end{array}$$

If we select *u*_0_ = *u*(*x*, 0) = cos⁡*x*, with the same process, we can also obtain the following exact solution of (20):

(25) $$\begin{array}{c} u=i\cos\left[\frac{x^{\beta }}{\Gamma \left(1+\beta \right)}\right]\text{Exp}\left[-\frac{1}{2}\frac{it^{\alpha }}{\Gamma \left(1+\alpha \right)}\right]. \end{array}$$

**Remark 8 .**

If one selects *β* = 1, the solution (23) is more standard than the result (5.10) in [23]. The solutions (23) and (25) are new exact solutions for (20) to our knowledge.

**Figure 1:**
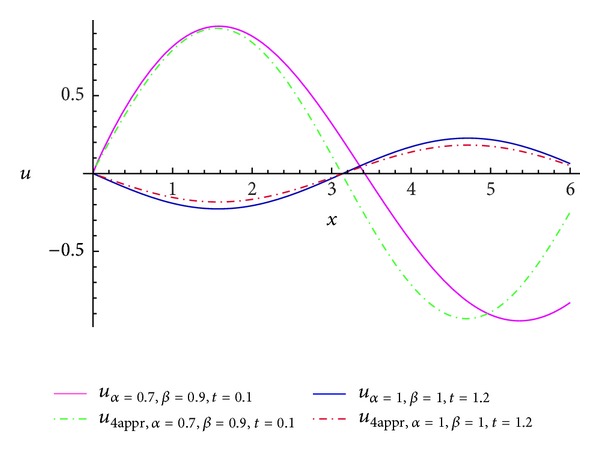
Comparison between the real part of *u*_4_ and the exact solution *u*.

**Figure 2:**
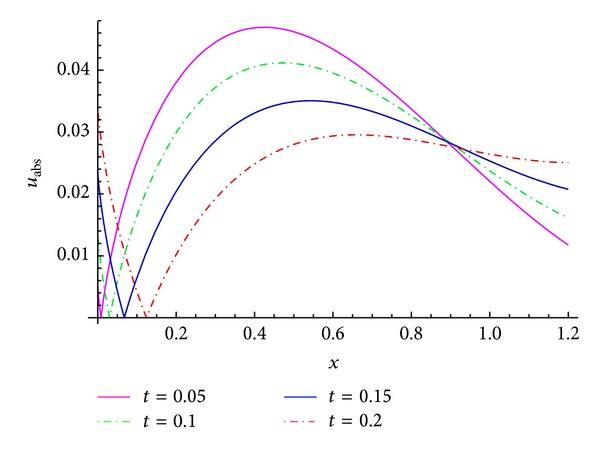
Plots of the absolute error *u*_abs_ when *α* = 0.7 and *β* = 0.9.

Comparisons between the real part of some numerical results and the exact solution (23) are summarized in Tables 1 and 2, and the simulations for *u*_4_, *u*_abs_, and *u* are plotted in Figures 1 and 2, which shows that the MFVIM produced a rapidly convergent series.

## 4. Summary

In this paper, the MFVIM is used for finding approximate and exact solutions of the GFNLS equation with Caputo derivative. The obtained results indicate that the MFVIM is effective, convenient, and powerful method for solving nonlinear fractional complex differential equations when comparing it with some other traditional asymptotic decomposition methods such as HAM, VIM, and ADM. We believe that these methods should play an important role for finding exact and approximate solutions in the mathematical physics.

## Figures and Tables

**Table 1:** Comparison between the real part of *u*_4_ and *u* when *α* = *β* = 1.

*x*	*t*	Approximate solution *u*_4appr_	Exact solution	Absolute error
1	0.4	0.6945501509	0.6944959727	0.0000541782
5	0.4	−0.7914960963	−0.7914343559	0.0000617404
1	0.3	0.7577097797	0.7577001100	9.66968 × 10^−6^
15	0.3	0.5855572741	0.5855498014	7.47272 × 10^−6^
12	0.2	−0.5126082301	−0.5126076876	6.16597 × 10^−6^
3	0.2	0.13481723570	0.13481709305	1.42655 × 10^−7^
2	0.1	0.8990870113	0.8990869969	1.43796 × 10^−8^
0.2	0.1	0.1964384915	0.1964384884	3.14175 × 10^−9^

**Table 2:** Comparison between the real part of *u*_4_ and *u* when *α* = 0.7, *β* = 0.9.

*x*	*t*	Approximate solution *u*_4appr_	Exact solution	Absolute error
1	0.4	0.5356787528	0.5565185584	0.02083980558
2	0.4	0.5092408025	0.6018550029	0.0926142003
1	0.3	0.6273816439	0.6535277868	0.02614614289
2	0.3	0.6242882934	0.7067670289	0.08247873543
1	0.2	0.7153235950	0.7417557951	0.02643220012
0.2	0.2	0.1978265247	0.2080382267	0.01021170194
0.2	0.1	0.1989280524	0.2288404399	0.02991238755
12	0.1	0.8741657132	0.8902825407	0.01611682753
